# A novel Granzyme B nanoparticle delivery system simulates immune cell functions for suppression of solid tumors

**DOI:** 10.7150/thno.35900

**Published:** 2019-10-14

**Authors:** Xiaomin Qian, Zhendong Shi, Hongzhao Qi, Ming Zhao, Kai Huang, Donglin Han, Junhu Zhou, Chaoyong Liu, Yang Liu, Yunfeng Lu, Xubo Yuan, Jin Zhao, Chunsheng Kang

**Affiliations:** 1 Department of Medical Laboratory, School of Medical Technology, Tianjin Medical University, Tianjin 300203, China; 2 3rd Department of Breast Cancer, China Tianjin Breast Cancer Prevention, Treatment and Research center, Tianjin Medical University Cancer Institute and Hospital, Tianjin 300060, China; 3 Tianjin Key Laboratory of Composite and Functional Materials, School of Materials Science & Engineering, Tianjin University, Tianjin 300072, China; 4 Department of Chemical and Biomolecular Engineering, University of California, Los Angeles, CA 90095, USA; 5 Laboratory of Neuro-Oncology, Tianjin Neurological Institute, Department of Neurosurgery, Tianjin Medical University General Hospital and Key Laboratory of Neurotrauma, Variation, and Regeneration, Ministry of Education and Tianjin Municipal Government, Tianjin 300052, China

**Keywords:** Granzyme B delivery, tumor therapy, nanoparticles, biomimetics

## Abstract

Cell-based immunotherapy for the treatment of hematologic malignancies, such as leukemia and lymphoma, has seen much success and played an increasingly important role in clinical studies. Nevertheless, the efficacy of immunotherapy in solid tumors still needs improvements due to the immunosuppressive properties of tumor cells and the microenvironment. To overcome these limitations, we prepared a novel tumor-targeting delivery system based on the underlying mechanism of immune-targeted cell death that encapsulated granzyme B protein within a porous polymeric nanocapsule.

**Methods**: A cell-penetrating peptide TAT was attached onto granzyme B (GrB) to enhance its transmembrane transport efficiency and potency to induce cell apoptosis. The endocytosis and internalization pathways of GrB-TAT (GrB-T) were analyzed in comparison with perforin by confocal microscopy and flow cytometry. Furthermore, the positively charged GrB-T was wrapped into nanoparticles by p-2-methacryloyloxy ethyl phosphorylcholine (PMPC)-modified HA (hyaluronic acid). The nanoparticles (called TCiGNPs) were characterized in terms of zeta potential and by transmission electron microscopy (TEM). The *in vitro* anti-tumor effects of GrB-T were examined by cell apoptosis assay and Western blotting analysis. The *in vivo* anti-tumor therapeutic efficacy of TCiGNPs was evaluated in a mouse tumor model.

**Results**: The TAT peptide could play a role similar to perforin to mediate direct transmembrane transfer of GrB and improve GrB-induced cell apoptosis. The TCiGNPs were successfully synthesized and accumulated in the solid tumor through enhanced permeability and retention (EPR) effect. In the tumor microenvironment, TCiGNPs could be degraded by hyaluronidase and triggered the release of GrB-T. The TAT peptide enabled the translocation of GrB across the plasma membrane to induce tumor cell apoptosis *in vivo.*

**Conclusion**: We successfully developed a granzyme B delivery system with a GrB-T core and a PMPC/HA shell that simulated CTL/NK cell-mediated cancer immunotherapy mechanism. The GrB delivery system holds great promise for cancer treatment analogous to the CTL/NK cell-induced immunotherapy.

## Introduction

The development of adoptive cell transfer therapy has revolutionized T cell-based immunotherapy for the treatment of cancers 1-3. For example, by isolating T cells from the peripheral blood of patients and remodeling them through genomic engineering, T cells can express a chimeric antigen receptor (CAR) or a new T cell receptor (TCR) and target and eventually kill cancer cells 4. To date, the success of T cell-based immunotherapy in treating hematological malignancies is impressive, particularly in infants, achieving up to 90% clinical response rates in acute lymphoblastic leukemia 5. Nevertheless, the clinical efficacy of T cells in solid tumors has been much less rewarding due to the poor penetration of immune cells into solid tumors 6-7. Also, the function of immune cells is inhibited even if they are present inside tumors due to the notorious hallmarks of the tumor microenvironment that include hypoxia and nutrient starvation 8-9.

An alternative way to treat solid tumors using T-cell therapy is to exploit their physiological function, mostly the granzyme-based mechanism. When cytotoxic T lymphocytes (CTLs) recognize target cells, granzymes are released from lytic granules and enter the cytoplasm of target cells where they efficiently induce cell apoptosis through direct and indirect caspase processing and activation, mitochondrial permeabilization, or targeting other nuclear proteins 10-11. Five different granzymes, A, B, H, K, and M, are found in humans, among which granzyme B is the most abundant type with high cytotoxic efficacy and a variety of apoptosis-inducing mechanisms 12. Over three hundred intracellular and extracellular human proteins have been identified as GrB substrates 13. In contrast with cell death-inducing cytokines of the tumor necrosis factor family, such as FASL and TRAIL, which require intact receptors and downstream signaling pathways to induce activation of initiator and effector caspases, cytosolic GrB can activate the apoptotic machinery directly ensuring induction of cell death even if other pathways are blocked 14-15.

Delivery of GrB has been demonstrated previously for anti-tumor therapy 16. GrB was fused with the single-chain antibody scFvMEL (anti-gp240), vascular endothelial growth factor 121, and transforming growth factor α (TGFα) and the data provided the first proof of concept that GrB-based anti-tumor agents could realize tumor inhibition *in vivo* 17. Recently nanoparticles have been shown to be efficient carriers for the delivery of GrB. For example, Zhong *et al*. entrapped GrB into galactose-decorated reduction-sensitive polymersomes and realized efficient suppression of hepatocellular carcinoma cells *in vitro* 18. Subsequently, they loaded GrB into Acupa-decorated pH-responsive chimeric polymersomes and efficiently delivered therapeutic proteins into prostate cancer cells 19. Recently, Liang *et al.* encapsulated GrB with hyaluronic acid-epigallocatechin gallate conjugates and linear polyethyleneimine self-assembled nanogel, which exhibited significant cytotoxicity to CD44-overexpressing HCT-116 colon cancer cells 20. These agents have provided encouraging results in GrB-based cancer treatments, but further improvement is needed to enhance its delivery efficiency *in vivo*, which is critical for translational tumor therapy in the clinic.

Endocytosis and direct translocation are the most common pathways of biomolecule uptake 21, 22. The endocytic pathway via the endosomal-lysosomal system is a classical defense mechanism against invasive viruses or pathogens 23. The internalized cargo, including genes and proteins delivered by the nano-vectors, undergoes acidification or degradation, resulting in low delivery efficiency and even delivery failure 24-25. As for GrB, its uptake by endocytosis is unavoidable even if it is fused with targeting factors or loaded in nanoparticles. Even worse, GrB is sensitive to most hydrolases rich in endosomes or lysosomes and might be degraded before it reaches the cytoplasm 26-27, resulting in relatively high IC_50_ values of GrB-based fusion proteins.

**Scheme 1:**
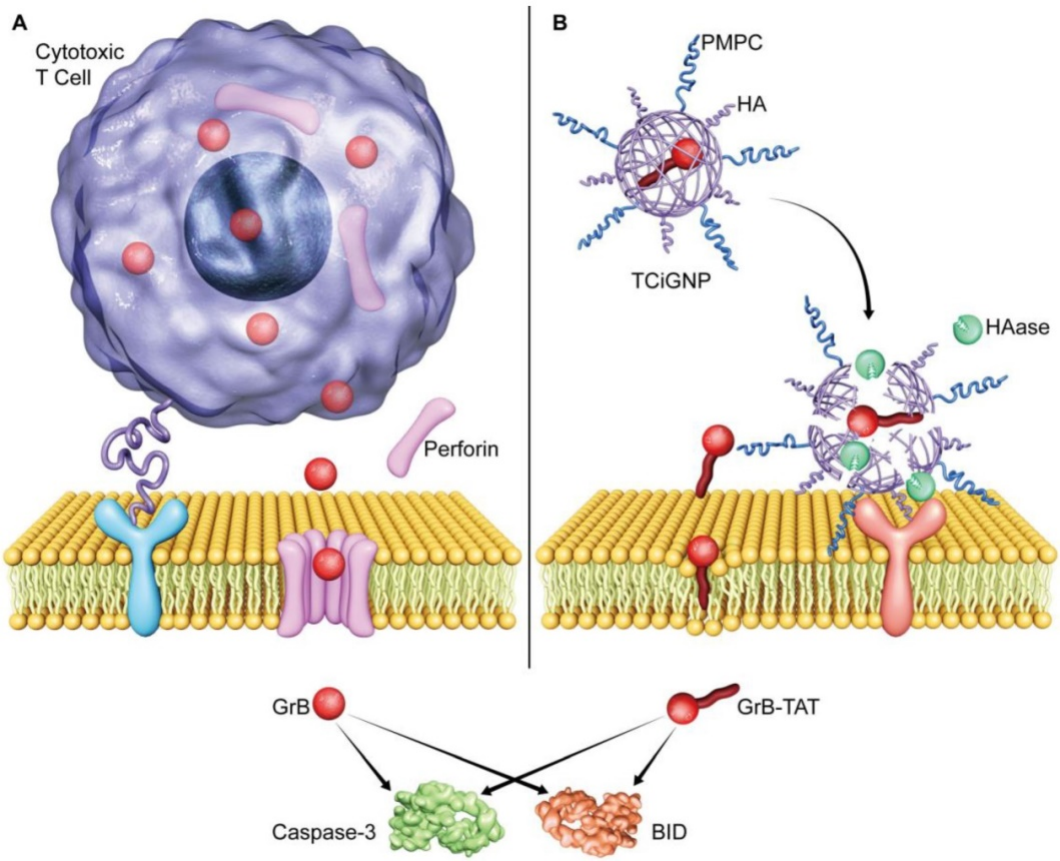
Illustration of GrB delivery system that functionally and mechanistically imitates the CTLs to kill target cancer cells. (**A**) CTLs deliver GrB directly to the cytosol *via* plasma membrane pores formed by perforin. (**B**) TCiGNPs have a GrB-T core and an HA/PMPC shell. The TAT peptide is assumed to play a similar role as perforin by inducing transmembrane transfer of GrB and improving GrB-mediated cell apoptosis. In the HAase-enriched tumor microenvironment, the HA/PMPC outer corona of TCiGNPs is degraded by HAase, followed by the extracellular release of GrB-T. On entry into the target cell cytosol, GrB-T promotes cell apoptosis via two main pathways, either through BID-dependent mitochondrial permeabilization or through direct caspase processing and activation, resulting in a significant anti-tumor effect.

In contrast to the endocytic route, the direct transmembrane entry into the cell is the key mechanism employed by biosomes for efficient delivery of biological factors to maximize their activity 28. For example, viruses fuse their envelope proteins with the host cell membrane, followed by forming a fusion pore on the surface, eventually releasing RNA or DNA into the host cell. In principle, GrB could also be delivered through this pathway. The CTLs release perforin and form pores on the surface of the target cell membrane, allowing direct diffusion of GrB into the cytoplasm of the cell thus helping its escape from endosome and lysosome degradation **(Scheme 1a)** 29-30. Consequently, abundant apoptosis pathways are activated, including caspase and BH3 interacting domain death (BID) agonist, and induce cell death (Scheme 1). However, since perforin is not stable and calcium-dependent, it is challenging to deliver exogenous GrB and perforin simultaneously into tumor cells 31-32.

Inspired by the CTL-mediated mechanism, we conceived a granzyme B delivery system that mimics the functionality of CTLs to deliver GrB and kill target cancer cells. As shown in **Scheme 1b**, GrB protein is conjugated with a cell-penetrating peptide (TAT), which functionally mimics the role of perforin, mediating the translocation of GrB into the cell cytosol. The positively charged GrB-T is wrapped into a nanoparticle with an average size of 33 nm by p-2-methacryloyloxy ethyl phosphoryl-choline (PMPC)-modified HA (hyaluronic acid). Once the nanoparticles (TCiGNPs) accumulate in solid tumors through enhanced permeability and retention (EPR) effect, HA would intrinsically target cancer cells because of CD44 expression on the cancer cells 33-34. However, due to the shielding effect of PMPC, the TCiGNPs linger on the cell surface until HA shells are degraded by hyaluronidase (HAase) overexpressed in the tumor microenvironment triggering the release of GrB-T 35. GrB would directly translocate across the plasma membrane with the help of TAT and induce tumor cell apoptosis. This TCiGNP system imitates the process of immune cells recognizing target cells and has shown considerable tumor suppression in an *in vivo* animal model.

## Materials and Methods

All chemicals were purchased from Sigma-Aldrich unless otherwise noted and were used as received. Granzyme B and perforin were obtained from Cloud-Clone Corp (Houston, USA). Sodium hyaluronic acid (HA, the molecular weight of 5kDa-150kDa) was acquired from TCI Development Co., Ltd. (Shanghai, China). TAT was purchased from ChinaPeptide. (Shanghai, China). Antibodies were obtained from Santa Cruz Biotechnology, Inc. Polyvinylidene fluoride (PVDF) membrane was acquired from Millipore, Inc.

### Synthesis of TCiGNPs encapsulating GrB in a PMPC/HA shell

GrB was dissolved in phosphate-buffered saline (PBS, PH 7.4) at 1mg/mL, followed by addition of an appropriate amount of succinimidyl 4-(N-maleimidomethyl)cyclohexane-1-carboxylate) (SMCC) and incubated for 2 hours at 4°C (GrB: SMCC,1:10, M: M). Subsequently, excess SMCC was removed using a desalting column equilibrated with conjugation buffer and TAT was added into the solution and further incubated for 2 hours at 4°C (SMCC: TAT: GrB,10: 10: 1, M: M: M). The resulting GrB-T was obtained by dialysis in PBS using centrifugal filters (10K MWCO) (Millipore). FITC-labeled GrB or GrB-T was synthesized by adding FITC to GrB or GrB-T (FITC-GrB-T,10:1) equilibrated in 0.2 M NaCl, sodium borate buffer, pH 9.2 for 2 h at ambient temperature. Uncoupled FITC was removed by dialysis in PBS for 48h (pH 7.4, Cut=10 kDa).

Cy5.5-labeled GrB-T was obtained by using the same method. HA-APM was obtained in two steps. First, HA, 1-Ethyl-3-(3-dimethylaminopropyl)-carbodiimide (EDC) and N-hydroxy-succinimide (NHS) were dissolved in MES buffer (PH 5.5) mixed for 1h (HA: EDC: NHS,5:5:2,M:M:M). The pH of the solution was adjusted to 8.0 using sodium hydroxide and hydrochloric acid, and subsequently, APM was added to the solution and mixed overnight at room temperature (unit HA: APM,1:1,M:M). The mixture was subjected to dialysis against PBS (Cut = 3.5 kDa) for 24h. Subsequently, GrB-T was added into HA-APM solution (GrB-T:HA-APM,1:1.2,W:W) followed by the MPC monomer (GrB-T:MPC,1:10000,M:M). Free-radical polymerization was then initiated by adding 1% ammonium persulfate solution (APS) and 1% N,N,N',N'-tetramethyl-ethylenediamine solution (TEMED) (MPC:APS:TEMED,20:1:2,M:M:M). The reaction was allowed to proceed at 4 °C for 2 h in a nitrogen atmosphere. Subsequently, the TCiGNPs were purified with hydrophobic interaction column (Phenyl-Sepharose CL-4B) to remove the unreacted protein 42 and dialyzed extensively at 4 °C in PBS using a 100-kDa membrane to remove monomers and initiators.

### Agarose gel electrophoresis

The agarose gel retardation was carried out in 1% (w/v) agarose gel in 1×TAE buffer at a constant voltage of 120 V for 15 min. The retardation of the nanoparticles was visualized at 365 nm using a UV gel image system (SIM135A, SIMON).

### Dynamic light scattering (DLS)

The sizes and zeta potentials of the TCiGNPs were determined by a zeta potential analyzer (Malvern Instruments, Worcestershire, UK) at 173° backscatter angle.

### TEM measurement

TCiGNPs were dropped onto a TEM carbon-coated copper grid (300 mesh) (Ted Pella) and then stained with 2% uranyl acetate for 2 min. After air drying, the sample was observed by TEMH-600 transmission electron microscope (Hitachi, Tokyo).

### Stability of TCiGNPs

500 μL of TCiGNPs were incubated at pH 7.4 in a 37ºC water bath or with serum. At predetermined time intervals, the particle sizes of the samples were measured by a Zetasizer (Malvern Instruments, Worcestershire, UK).

### Degradation of HA and *in vitro* HAase--mediated GrB-T release

500 μL of TCiGNPs and GrB-T/HA were incubated with HAase at pH 6.5 in a 37 ºC water bath (HA: Hyase, 1:3,w:w). At predetermined time intervals, the particle size and zeta potential of the samples were measured by a Zetasizer (Malvern Instruments, Worcestershire, UK). Also, at various time intervals, free GrB-T was harvested using centrifugal filters (60K MWCO) (Millipore). The fluorescence intensity of GrB-T was determined at 280 nm with a UV-Vis spectrometer (Bechman Coulter DU®730 UV/Vis).

### Cell culture conditions

MDA-MB-231 and U87cells were purchased from China Academia Sinica Cell Repository (Shanghai, PR China). Macrophage cells J774A.1 were purchased from the American Type Culture Collection (ATCC). MDA-MB-231 and U87 cells were cultured in RPMI-1640 medium and DMEM, respectively, with 10% fetal bovine serum at 37 °C in a humidified atmosphere containing 5% CO2. The cells were sub-cultured at 70~80% confluence.

### Cell uptake

MDA-MB 231 cells (2×10^5^) were seeded into a 6-well plate containing coverslips in the wells and cultured for attachment. FITC-labeled GrB(50nm/L), GrB-T, GrB/PFN, TCiGNPs, and TCiGNPs treated with HAase for 6h were added to the cells and incubated for 4h at 37 °C. Subsequently, the cells were washed three times with PBS and fixed in 4% paraformaldehyde in PBS for 15 min. Then, the cells were incubated with the LysoTracker Blue (50 nM, Molecular Probe, Invitrogen Co, OR, USA) for 0.5 h for endosome/lysosome labeling. The cells were then washed three times with PBS and observed using CLSM (Carl Zeiss Microscope systems, Jena, Germany).

For uptake efficiency analysis, MDA-MB 231 cells (1×10^5^) and J774A.1 cells (1×10^5^) were seeded into a 24 well plate and incubated with various formulations of GrB as described above for 4h at 37°C. Subsequently, the cells were harvested and analyzed by flow cytometry (BD FACS Calibur). J774A.1 cells were observed by fluorescence microscopy.

To explore the uptake pathways for GrB-T and GrB/PFN, several membrane entry inhibitors including chlorpromazine (CPZ), amiloride (Ami), methyl-b-cyclodextrin (MBCD), and lovastatin were added to cultured MDA-MB-231 cells for 30 min pre-treatment observed using CLSM 36. Each test was repeated three times.

### Transwell migration assay

MDA-MB-231-coated Transwell assay was performed as previously described 38. Prior to the introduction of the GrB and GrB-T, the medium in the basolateral compartment of the Transwells was replaced with the medium for the transcytosis assay (IPMI-1640 without phenol red, 5% FBS and 2% penicillin/streptomycin), to reduce the background for fluorescence intensity measurement. 100 μL of FITC-GrB and FITC-GrB-T (50nm/L) was added into the apical compartment at 37°C for 24 h, and the fluorescence intensity of basolateral compartment was analyzed at various time points by a plate reader.

### *In vitro* cytotoxicity

MDA-MB-231 and U87 cells (5 × 10^3^ cells/well) were seeded in 96-well plates and cultured for 24 h. The cells were exposed to various GrB formulations at different concentrations of GrB for 48 h. 10 μL of the resazurin sodium salt solution (0.1%) was added to each well and the cells were stained for 4 h. The fluorescent signal was monitored using 530-560nm excitation wavelengths and 590 nm emission wavelength by a microplate reader (Fujifilm BAS-5000 Infinite M200 PRO, Tecan). Each test was repeated three times.

### Cell apoptosis assay

Apoptosis of MDA-MB-231 and U87 cells were detected using the Annexin V-FITC Apoptosis Detection Kit (BD Biosciences). The cells (1 × 10^5^ /well) were seeded in 6-well plates and cultured for 24 h. Then the cells were incubated with various GrB formulations as described for the *in vitro* cytotoxicity assay with GrB 100nm/L for 48h. Finally, the cells were analyzed by flow cytometry (BD FACSCalibur) according to the manufacturer's protocol. Each test was repeated three times.

### Protein extraction and Western blotting analysis

MDA-MB-231 cells were treated with various GrB formulations as shown in the cell apoptosis assay. After 48h, each group of cells was washed with PBS three times and then solubilized in 1% of Nonidet P-40 lysis buffer. Then, total proteins were extracted, and Western blot analyses were conducted according to the manufacturer's instructions as previously described 37-38. GAPDH was used as a housekeeping gene. The proteins were detected using a Super Signal protein detection kit (Pierce, Rockford, IL). Each test was repeated three times.

### Animals and tumor xenograft models

BALB/c-A nude mice at about 4-6 weeks of age were purchased from the animal center of the Cancer Institute of Chinese Academy of Medical Science. All animals were treated in accordance with the Guidelines for Care and Use of Laboratory Animals, approved by Institutional Animal Care and Use Committee. MDA-MB-231 breast model was established in BALB/c-A nude mice according to the method described before 39. The tumor volume was measured using the formula: volume = length×width^2^/2.

### *In vivo* imaging study and tumor distribution

When the tumors reached 200 mm^3^, the mice (n=6) were intravenously injected with Cy-5.5-GrB, Cy5.5-GrB-T, and Cy5.5-TCiGNPs. Mice were imaged using bioluminescence imaging at 24, 48, and 96 h post-injection.

FITC labeled GrB, GrB-T, and TCiGNPs were intravenously injected as the Cy-5.5-labeled samples. Tumors were removed from the mice for consecutive preparation of frozen sections of 5 mm thicknesses. Nuclei were stained with DAPI. The distribution of fluorescence was observed by a confocal microscope (Carl Zeiss Microscope systems, Jena, Germany).

### Protein adsorption assay

10μL each of PBS (negative control), GrB (1mg/mL), GrB-T, and TCiGNP was mixed with 30 μL of mouse whole serum and incubated in a shaker at 37 °C for 30 min. After incubation, all samples were filtered and washed with PBS for 3 times with centrifugal filtration (molecular weight cut-off, MW=100 kDa) to remove unabsorbed serum proteins. Subsequently, samples were reconstituted with 50 μL of PBS and the amount of GrB in each sample was measured using the BCA Protein Assay kit (Thermo, USA) and BSA as the standard. Finally, the amount of protein adsorbed was determined by measuring the overall protein concentration of each sample using the BCA assay. Each test was repeated three times.

### * In vivo* anti-tumor efficacy

When the tumors reached 100 mm^3^, mice (n=4) were intravenously injected with saline, GrB, GrB-T, and TCiGNPs every three days. The tumor size and body weight of the mice were measured. At Day 37, the tumors were harvested from the mice after euthanasia, washed with saline thrice and then fixed in 10% neutral buffered formalin (NBF). The paraffin-embedded tissue sections were used for HE staining and immunohistochemical staining as previously described 40. The comparative survival of the tumor-bearing animals (n=6) was assessed with Kaplan-Meier curves based on the Kaplan-Meier estimator.

## Results and Discussion

### Translocation across the plasma membrane and inhibition of proliferation by GrB-T

**Figure 1:**
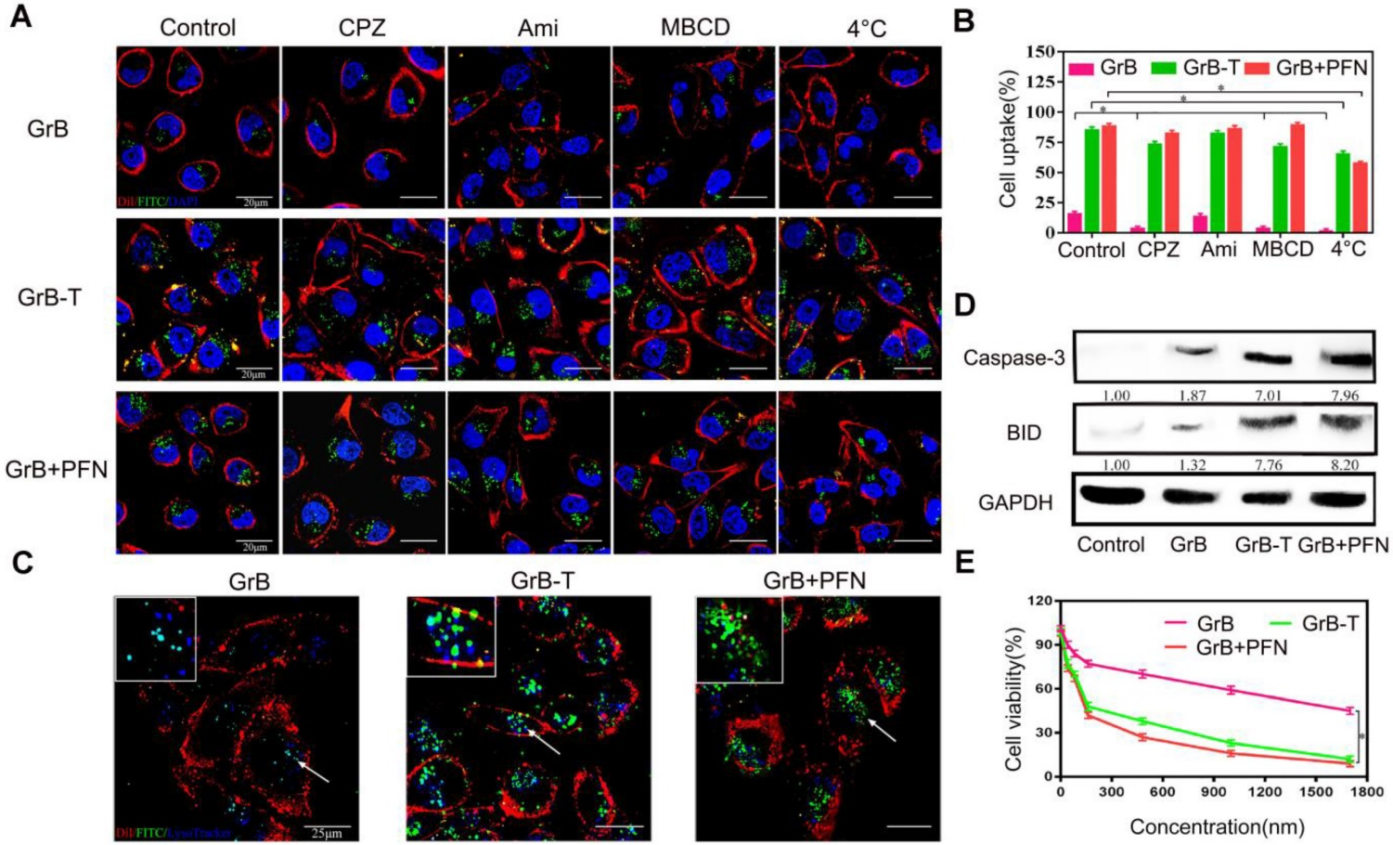
(**A**, **B**) Cell uptake of GrB, GrB-T, and GrB/PFN(GrB+PFN) by confocal microscopy and flow cytometry following treatment with different endocytosis inhibitors *P<0.05. The nuclei and the cell membranes were stained with DAPI (blue) and DiI (red), respectively. (**C**) Colocalization of various reagents in MDA-MB-231 cells by confocal microscopy. The lysosomes were stained with LysoTracker Blue. The arrows indicate the fluorescence of GrB.(**D**) Caspase-3 and BID protein expression by Western blotting in MDA-MB-231 cells treated with various reagents. (**E**) Cell viability detected by resazurin with indicated treatments.

To verify that TAT could mimic perforin to deliver GrB across the cell membrane directly, the cellular uptake mechanism of GrB-T was studied by endocytosis inhibition. The results were compared with that of GrB/perforin (GrB/PFN). MDA-MB-231 cells were incubated with FITC-labeled GrB, GrB-T, or GrB/PFN for 4 hours with or without endocytosis inhibitors, followed by nuclear and cell membrane staining by DAPI and Dil, respectively. As shown in **Figure 1a** and **1b**, GrB could enter the cell through receptor-mediated endocytosis 41. When cells were pre-treated with CPZ (inhibitor of clathrin-dependent endocytosis), or MBCD (inhibitor of caveolin-dependent endocytosis), the uptake of GrB was significantly decreased. Moreover, conjugation with TAT or PFN significantly enhanced the uptake of GrB by MDA-MB-231 cells. Analogous to the PFN-mediated internalization, neither CPZ nor MBCD affected the cellular uptake of GrB-T. Also, when cultured cells were pre-incubated with amiloride (50 μM), a specific inhibitor of the Na^+^/H^+^ exchange required for macropinocytosis, there was no change in GrB uptake compared with the uptake by untreated cells as well as cells treated with GrB-T and GrB/PFN.

Thus, the inhibition studies suggested that the TAT peptide guides GrB to enter cells through a non-endocytic mechanism. To prove the effect of TAT penetrating peptide, translocation of the GrB-T through breast cancer cell MDA-MB-231 was performed in a Transwell assay 42 with GrB as a control. As shown in **Figure S1**, the penetration of GrB-T increased with incubation time and reached 23% after 8h. For comparison, GrB without TAT showed a much lower penetration efficiency of 2.1%. With prolonged culture time, the penetration efficiency of GrB-T increased to 29.5% after 24h of incubation, while the GrB group reached 3.59% confirming the penetrating ability of TAT peptide.

Since clathrin- and caveolin-dependent endocytosis is contingent on ATP 43, we measured temperature-dependent uptake of GrB. As shown in **Figures 1a** and **1b**, cooling cells to 4 °C suppressed the internalization of GrB, whereas a remarkable fluorescence intensity of GrB-T and GrB/PFN was observed, further demonstrating the translocation mechanism of TAT- and perforin-mediated GrB uptake by MD-MBA-231 cells.

The different uptake pathways of GrB led to discrete intracellular fates, and also affected its inhibition of tumor cells 44. When GrB alone entered the tumor cells through the endocytosis pathway, it was captured by the endosome and lysosome (**Figure 1c**), and mainly degraded by proteases. As determined by flow cytometry, under the same cell uptake conditions, the ability of GrB to regulate its downstream targets, caspase-3 and BID, was significantly lower than that by GrB-T and GrB/PFN (**Figure 1d**). These results demonstrated that TAT and perforin could transport GrB directly into the cytoplasm bypassing the endosomal and lysosomal capture and thereby significantly enhanced the expression of caspase-3 and BID to induce cell apoptosis (**Figure 1d**). As expected, compared with GrB treated cells, the cell viability decreased sharply upon treatment with GrB-T or GrB/PFN (**Figure 1e**). The half-maximal inhibitory concentration (IC50) of GrB-T, which was slightly higher than GrB/PFN, showed 11.5-fold increase in cytotoxicity compared to GrB without TAT decoration, demonstrating that TAT promoted efficient cell proliferation inhibition. The above results indicated that TAT could functionally mimic perforin-mediated GrB transmembrane transport into tumor cells and induce cell apoptosis.

### Preparation and characterization of TCiGNPs

**Figure 2:**
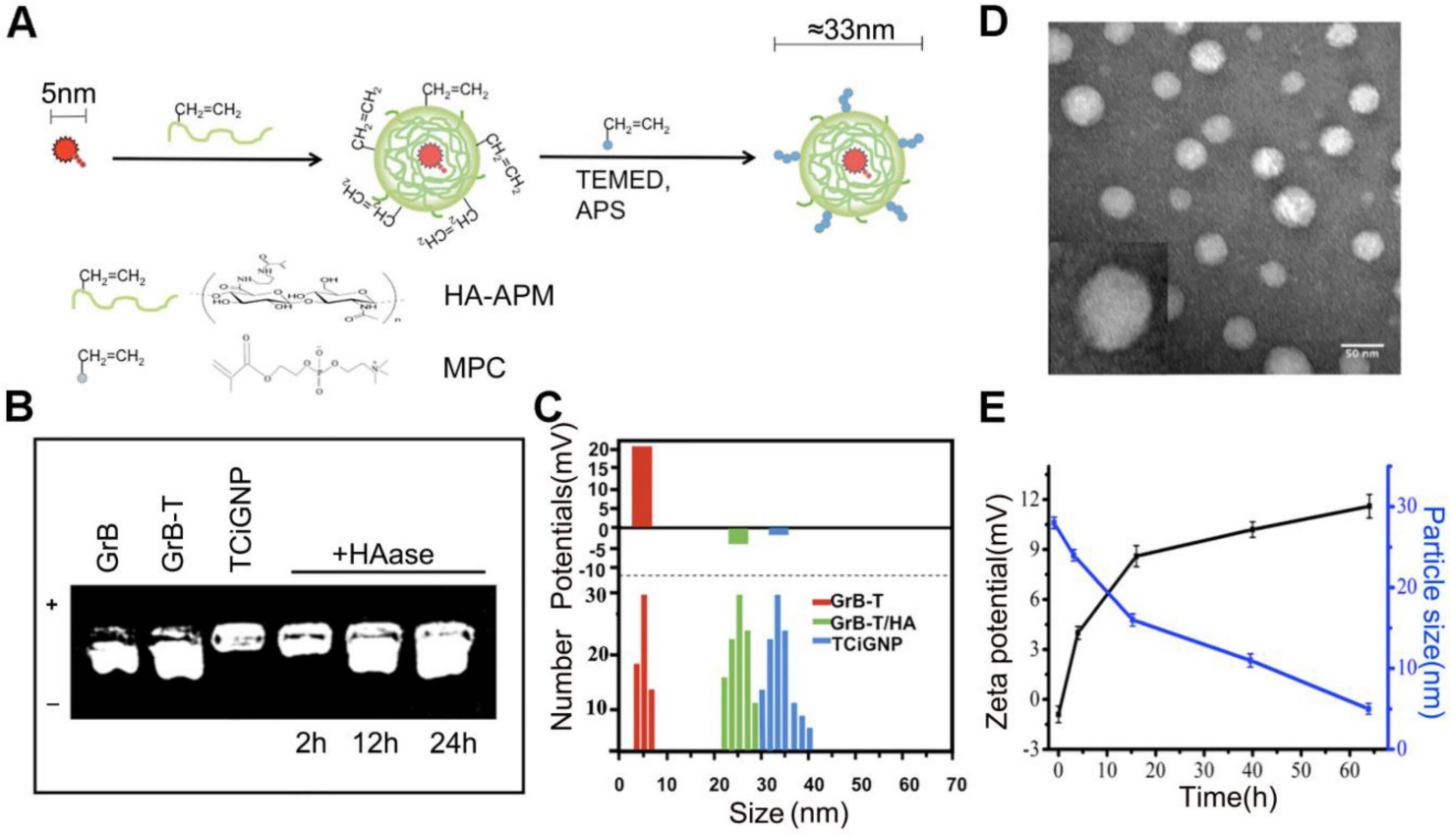
(**A**) Design procedure of TCiGNPs for site-specific GrB delivery. (**B**) Agarose gel electrophoresis of various GrB formulations. (**C**) Particle size and zeta potential of GrB-T, GrB-T/HA, and TCiGNP.(**D**) TEM images of TCiGNPs.(**E**) Change in particle size and zeta potential of TCiGNPs over time incubated with HAase at pH 6.5.

To facilitate the delivery of GrB-T *in vivo* and ensure its extracellular release, the GrB-T was complexed with HA-APM (Hyaluronic acid- N-(3-aminopropyl) methacrylamide) via electrostatic interaction, following which MPC was polymerized on the surface of the GrB-T/HA-APM complexes to form TCiGNPs (**Figure 2a**). HA-APM synthesis was demonstrated by 1H NMR (**Figure S2**) and the successful joining of TAT to GrB (**Figure S3**). The formation of TCiGNPs was monitored by agarose gel electrophoresis, and size analysis and zeta potential measurement were carried out. As shown in **Figure 2b**, both GrB and GrB-T showed a positive charge (14 mV and 25 mV, respectively) moving to the anode under the electric field. When GrB-T was attached to HA-APM, the (GrB-T/HA) particle size of the complex increased to 25 nm, but its zeta potential decreased to -4 mV (**Figure 2c**). Finally, PMPC was coated on the surface of GrB-T/HA complex through *in situ* polymerization forming TCiGNPs with a uniform diameter of 33 nm and a nearly neutral zeta potential of -1.3mV (**Figure 2c**), suggesting complete encapsulation of the protein. The anti-fouling property of PMPC was expected to significantly reduce the non-specific interaction between TCiGNPs and cancer cells, thereby avoiding protein-mediated cell endocytosis 45,46. The transmission electron microscope (TEM) image (**Figure 2d**) of TCiGNPs showed that the nanoparticles were spherical, and the particle size was consistent with the light scattering test results.

To verify the degradation susceptibility of the HA-PMPC shell by HAase abundant in the tumor microenvironment triggering the release of GrB-T, TCiGNPs were incubated with 0.6 μg/mL HAase, a concentration similar to that of tumor 47. The release of GrB-T was evaluated by agarose gel electrophoresis. As displayed in **Figure 2b**, incubation of TCiGNP with HAase for 12 and 24 h generated a well-defined band identical to that of GrB-T, demonstrating the ability of HAase to trigger the release of positively charged GrB-T from TCiGNPs. The results were confirmed by the particle size and zeta potential measurements as well. The particle size reduced sharply from 35 nm to 15 nm during the first 15 h, while the surface charge of the nanoparticles reversed from -1.3 mV to +9 mV (**Figure 2e**), suggesting that the HA-PMPC shell degradation led to the exposure of positively charged GrB-T. In contrast, in the absence of HAase, no GrB-T was released from the TCiGNPs. The HAase-mediated release of GrB-T was also evaluated using centrifugal filters. **Figure S4** shows that only 4.8% of GrB-T was released from TCiGNPs in the first 4h and about 7.5% was released within 12h in the absence of HAase. However, the presence of HAase accelerated the release of GrB-T from TCiGNPs. After incubation with HAase, 28% of GrB-T was released from TCiGNPs in the first 4h and more than 65% was released within 12h. The nanoparticles kept their size and no obvious aggregation occurred within 5 days in PBS at 37C° (**Figure S5**), indicating excellent stability of the nanoparticles. Furthermore, even after a week of mixing with serum at 37C° (**Figure S6**, Supporting Information), there was no noticeable change in TCiGNP particle size, proving good stability of TCiGNPs after entering the blood circulation.

### Extracellular delivery of GrB-T by TCiGNPs and translocation across the membrane

**Figure 3:**
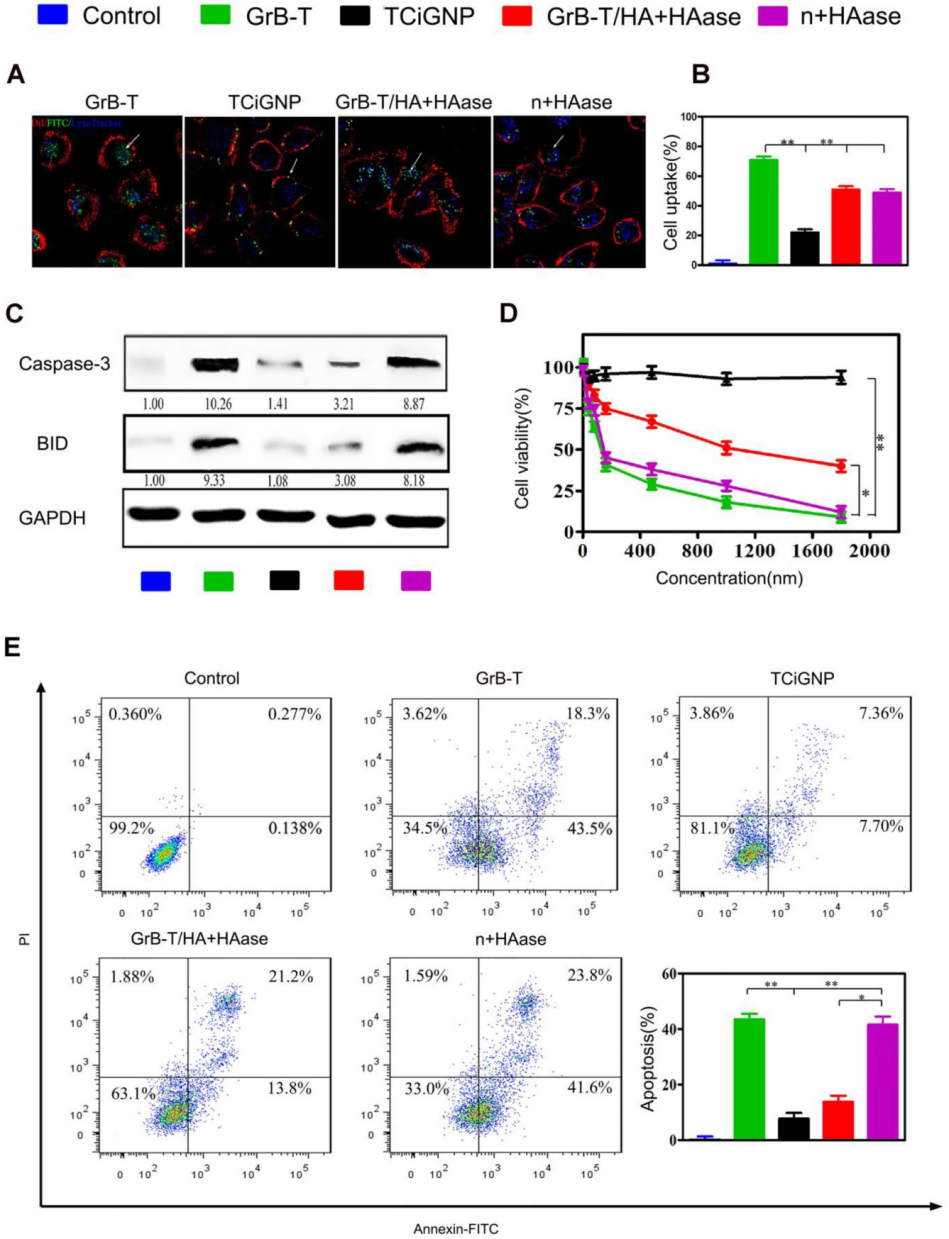
(**A**, **B**) Uptake of the TCiGNPs and HAase-pre-treated TCiGNPs (n+HAase) in MDA-MB-231 cells by confocal microscopy. The arrows indicate the fluorescence of the GrB. (**C**) Caspase-3 and BID protein expression in MDA-MB-231 cells treated with various GrB formulations as indicated. (**D**) MDA-MB-231 cells treated with GrB formulations detected by resazurin. (**E**) Apoptosis in MDA-MB-231 cells treated with GrB formulations detected by flow cytometry. Scale bars are 25 μm. *P<0.05, **P<0.01.

To test whether TCiGNPs could extracellularly deliver and translocate GrB through the plasma membrane, human breast cancer cells (MDA-MB-231) were incubated with TCiGNPs with or without HAase pre-treatment; GrB-T and GrB-T/HA were used as controls. As seen in the CLSM images (**Figures 3a** and **3b)**, GrB-T/HA could enter cancer cells when incubated with HAase for 6h. The internalization pathway, however, was different from that of GrB-T, which entered the cells through a non-endosome pathway (**Figure 1a**), while most of the GrB-T/HA fluorescence coincided with the endosome fluorescence (**Figure 3a**). This could be explained by the fact that HA was difficult to be degraded by HAase in a short time and most GrB-T/HA particles were still intact (**Figure S4**). However, HA could still guide GrB-T through the endosome-dependent pathway into the cells by binding to the CD44 surface receptors of MDA-MB-231 cells. Unlike GrB-T/HA, TCiGNPs could not enter the cells without HAase treatment and therefore bound on the surface of the cell membrane (**Figure 3a**) resulting from the anti-fouling function of MPC. Nevertheless, after HAase treatment, most GrB-T fluorescence was seen in the cell cytoplasm via the non-endosome pathway (**Figure 3a**).

It was previously reported that high hydrophilicity of the PC group inhibited non-specific protein adsorption on the nanoparticle surface and receptor-mediated cell uptake 48. Likewise, in this study, TCiGNPs were resistant to cellular uptake due to the presence of a stable and chilled water layer that inhibited the interface interaction between cells and TCiGNPs allowing sufficient time window for HA degradation by HAase. Subsequently, the released GrB-T from the TCiGNPs adhered on the cell surface for direct translocation into the cell (**Figure 3a and 3b**). In such a scenario, GrB activity could be maintained to realize better cancer cell inhibitory effects (**Figure 3d and Figure S7a**). Once delivered into the cytoplasm, GrB-T induced apoptosis by directly activating caspases-3 in the cytosol and cleavage of BID in the mitochondria through cell death pathways. The expression levels of these two proteins in MDA-MB-231 cells were examined by Western blotting (**Figure 3c**). The results showed that GrB-T and TCiGNPs with HAase pre-treatment resulted in significantly increased expression of caspase-3 and BID while other cells used as controls maintained their stable expression, which was consistent with the cell apoptosis assay (**Figure 3e** and **Figure S7b**).

Although TAT was beneficial for the escape of GrB-T/HA from endosome and lysosome, the endosome pathway still affected the ability of GrB to induce apoptosis compared with the direct transmembrane pathway. This extracellular delivery of GrB-T into tumor cells simulated the functioning of CTLs, and a smaller particle size of TCiGNP nanoparticles was more conducive to targeting solid tumors, response to HAase release, and intracellular delivery of GrB-T.

### *In vivo* delivery of TCiGNPs and suppression of tumors

Most importantly, this study aimed to deliver GrB into the immunosuppressive tumor microenvironment and inhibit tumor growth. To achieve this goal, the MDA-MB-231 tumor-implanted nude mice, which were able to express PDL-1 receptors with immunosuppressive properties 49, were used as the tumor-bearing animal model. Immunohistochemistry demonstrated high expression of PDL-1 protein on the tumor surface (**Figure S9**), inhibiting the recognition and killing functions of immune cytotoxic T cells within the tumor microenvironment, leading to the failure of cellular immunotherapy.

**Figure 4:**
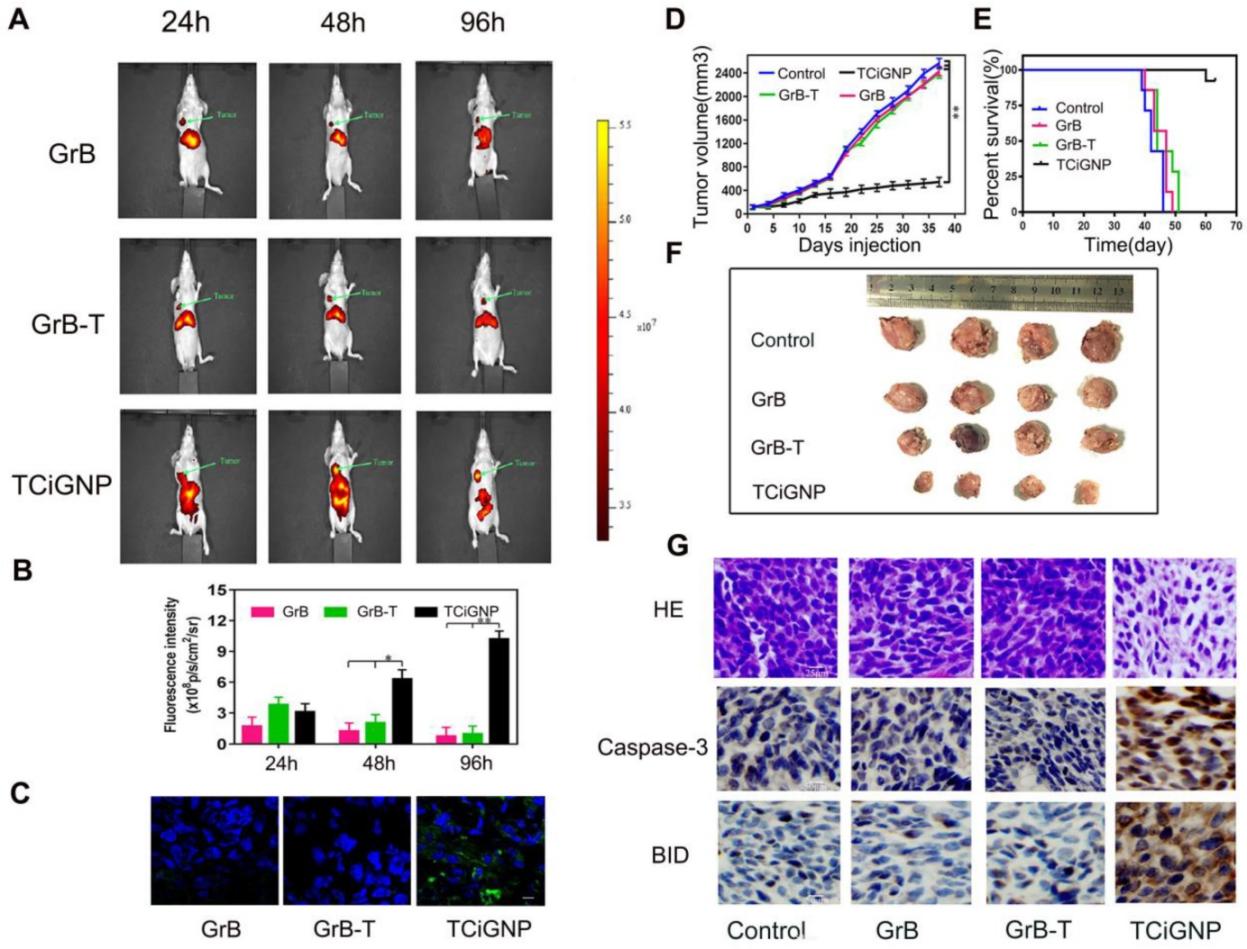
(**A**, **B**). Nanoparticle distribution in nude mice detected by an *in vivo* imaging system (**C**) Immunofluorescence staining of the tumors 96 h after treatment with TCiGNPs compared to the free GrB and GrB-T (red: tumor, blue: nucleus, green: GrB) (**D**, **F**). Tumor volume change at different times in different groups. (**E**). Survival rates of mice (n=7) by the Kaplan-Meier method. (**G**). H&E staining detected the tumor organizational structure in different groups. Caspase-3 and BID expression in different groups were analyzed through immunohistochemistry. Scale bars are 50 μm *p<0.05.**p<0.01.

TCiGNPs exhibited high accumulation in the tumor after systemic administration due to their small size, prolonged blood circulation, and surface incorporation of MPC oligopolymer 50 that suppressed serum protein adsorption (**Figure S10**) and macrophage uptake (**Figure S8**). As shown in **Figure 4a**, GrB and GrB-T, due to cationic charges, were mainly concentrated in the liver 24 h post intravenous injection. Although a small amount of GrB and GrB-T aggregates could be found at the site of the tumor, their intensities in the tumor rapidly decreased over time (**Figure 4b**). In contrast, TCiGNPs initially accumulated at the tumor site in amounts comparable to GrB-T, but as time extended, a higher fluorescence signal was observed in the tumor region compared with that in the normal tissues at 96 h post-injection (**Figures 4a and b**). The accumulation of TCiGNPs in the tumor cells 96h post-injection was further confirmed (**Figure 4c**), suggesting that TCiGNPs could mediate GrB entry into tumor cells, induce tumor cell apoptosis, and inhibit tumor growth.

As is evident from **Figure 4d and f**, tumor growth was remarkably suppressed after the successive intravenous injections of TCiGNPs compared with saline, GrB, and GrB-T negative control groups. Most significantly, the TCiGNP nanoparticles markedly improved mice survival rate over the PBS control. At day 51, all mice were alive in the TCiGNP group (**Figure 4e**), whereas there was no change in survival rates in GrB or GrB-T groups relative to PBS, indicating that GrB induced little therapeutic effect. The hematoxylin and eosin (HE) staining of the tumor sections showed a massive cancer cell remission and decreased nuclear staining after TCiGNP treatment (**Figure 4g**), providing the most convincing evidence of the anti-tumor efficiency of TCiGNPs *in vivo*. The expression of caspase-3 and BID, which are important GrB substrates 51, was also determined at the molecular level by the representative photomicrographs of immunohistochemistry. As shown in **Figure 4g**, the expression of caspase-3 and BID was significantly increased in the TCiGNP-treated group compared with other groups.

In summary, our results confirmed tumor suppression *in vivo* following GrB-T release from TCiGNPs. Furthermore, we compared the GrB IC50 and apoptosis and survival rate of TCiGNPs with already reported particles 52-54 (**Table S1**). The comparison displayed that TCiGNPs showed remarkable therapeutic efficacy and significantly prolonged the survival time of mice. Taken together, it was verified that TCiGNPs preferentially accumulated at the tumor site, efficiently delivered GrB-T to the specific sites of activity, and consequently accomplished promising anti-tumor efficacy.

## Conclusion

We successfully developed a granzyme B delivery system with a GrB-T core and an HA/PMPC shell for site-specific tumor treatment that simulated CTL/NK cell-mediated cancer immunotherapy. The TAT peptide could play a similar role as perforin to induce GrB direct transmembrane translocation and improve GrB-induced cell apoptosis. Like CTL/NK cells, the HA/PMPC outer corona of TCiGNPs was degraded by HAase, in the HAase-enriched tumor microenvironment, followed by the extracellular release of GrB-T to enter cell cytoplasm and trigger subsequent extrinsic apoptosis pathways, resulting in a significant anti-tumor effect. Besides GrB, other immunotoxin-like proteins could also be encapsulated into the HA/PMPC shell and released in response to HAase, providing ample opportunities for cancer therapy. In conclusion, the GrB delivery system holds great promise for cancer treatment analogous to the CTL/NK cell-induced immunotherapy.
