# Supplementary material for: A novel Granzyme B nanoparticle delivery system simulates immune cell functions for suppression of solid tumors
Source: Theranostics. 2019 Oct 14;9(25):7616–27. doi: 10.7150/thno.35900 (PMC6831455; doi:10.7150/thno.35900)
Supplement: Supplementary file 1 — Supplementary figures and tables. [file thnov09p7616s1.pdf]

Supplementary Materials
Supplemental Figures

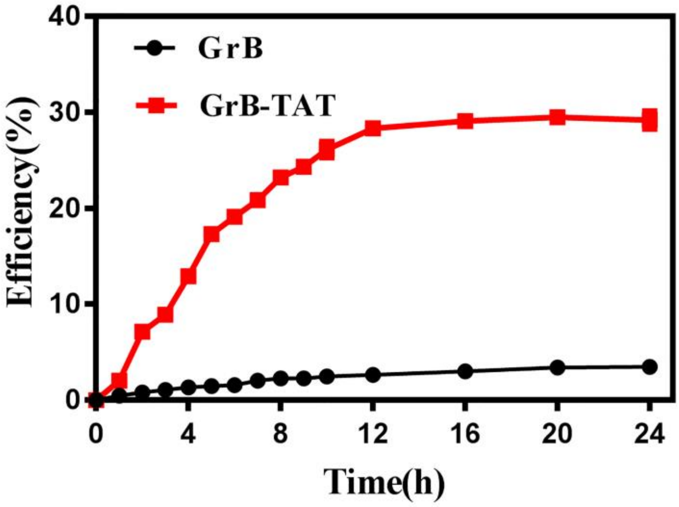

Figure S1. Accumulative penetration efficiency of GrB-T labeled with FITC through MDA-MB-231 cells by Transwell assay. The fluorescence intensity in the basolateral compartment was monitored.

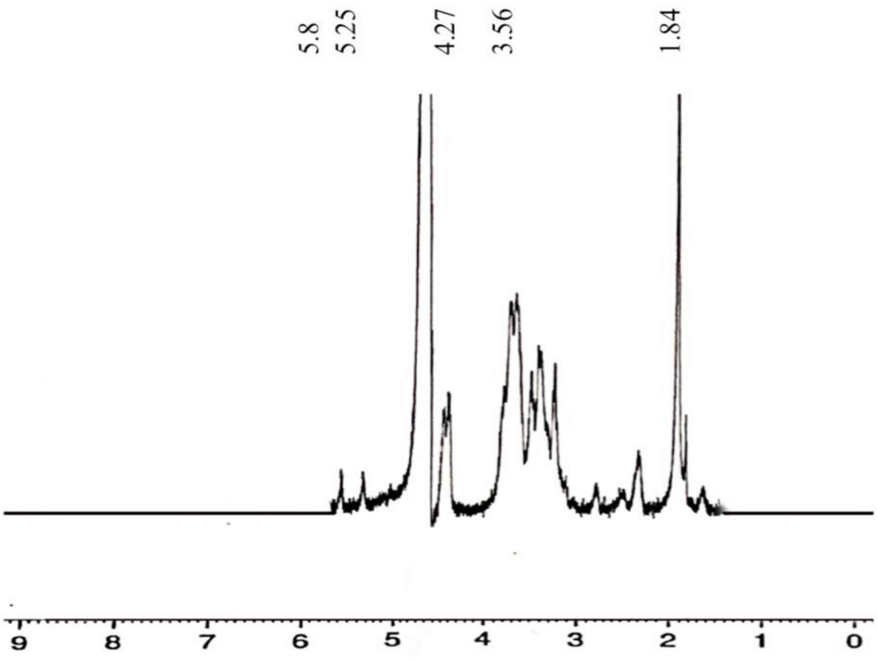

Figure S2. 1H-NMR spectrum of HA-APM

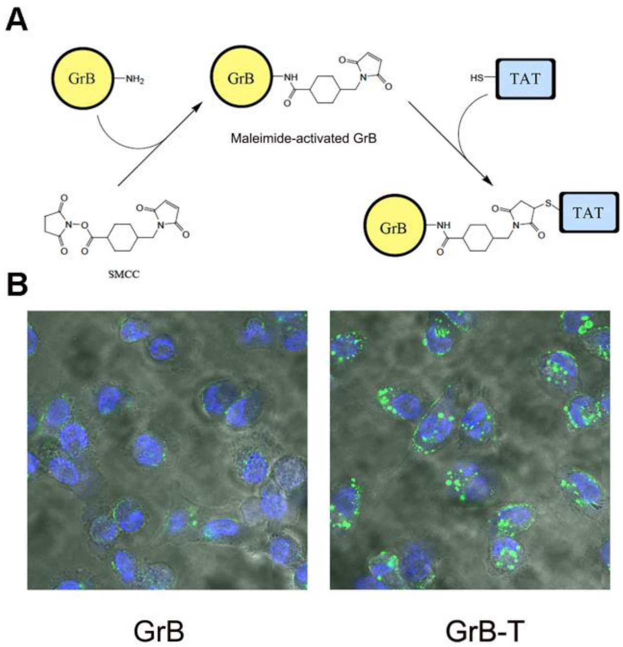

Figure S3. (**A**) Schematic of GrB and TAT connection through SMCC. (**B**) Uptake of the GrB and GrB-T in MDA-MB-231 cells by fluorescence microscopy. GrB was labeled by FITC and the nuclei were stained by DAPI. Scale bars are 20 μm.

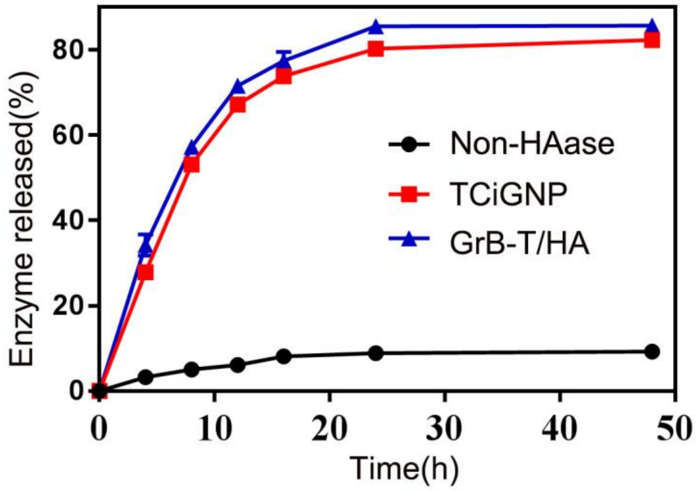

Figure S4. GrB-T release from TCiGNPs and GrB-T/HA degraded by Hyaluronidase.

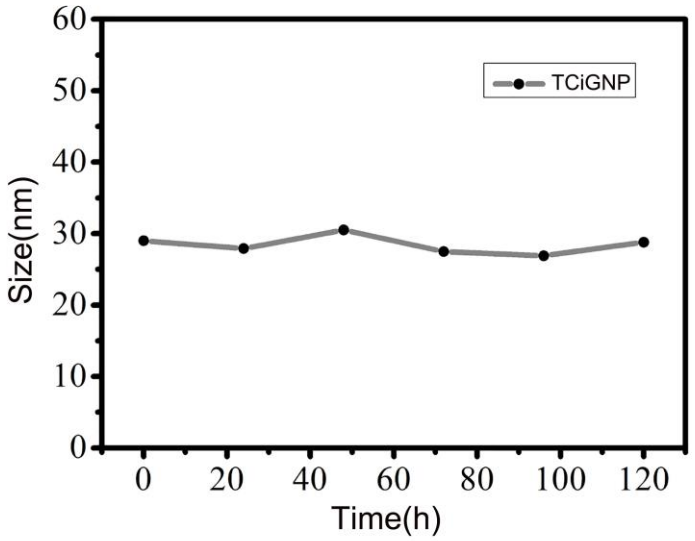

Figure S5. Change in particle size of TCiGNPs incubated for 5 days in PBS at 37C° over time.

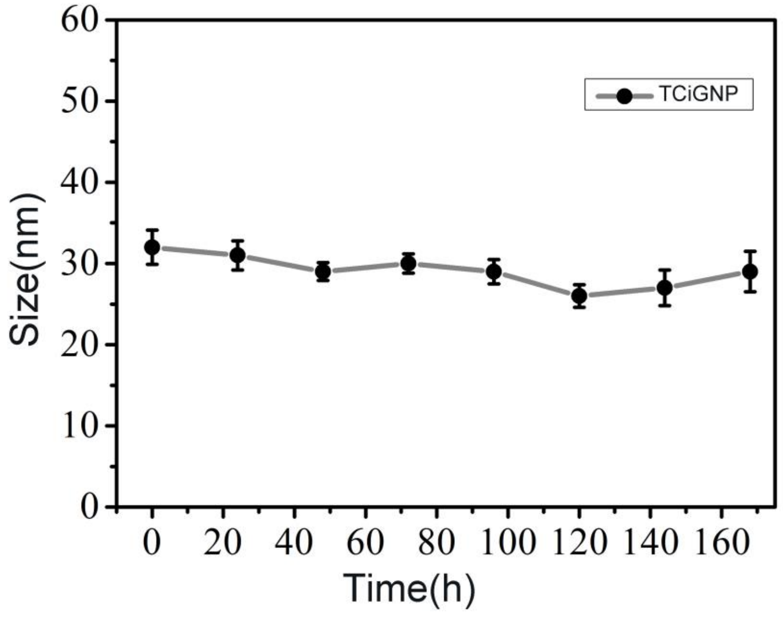

Figure S6. Change in particle size of TCiGNPs incubated for 7 days at 37°C in serum.

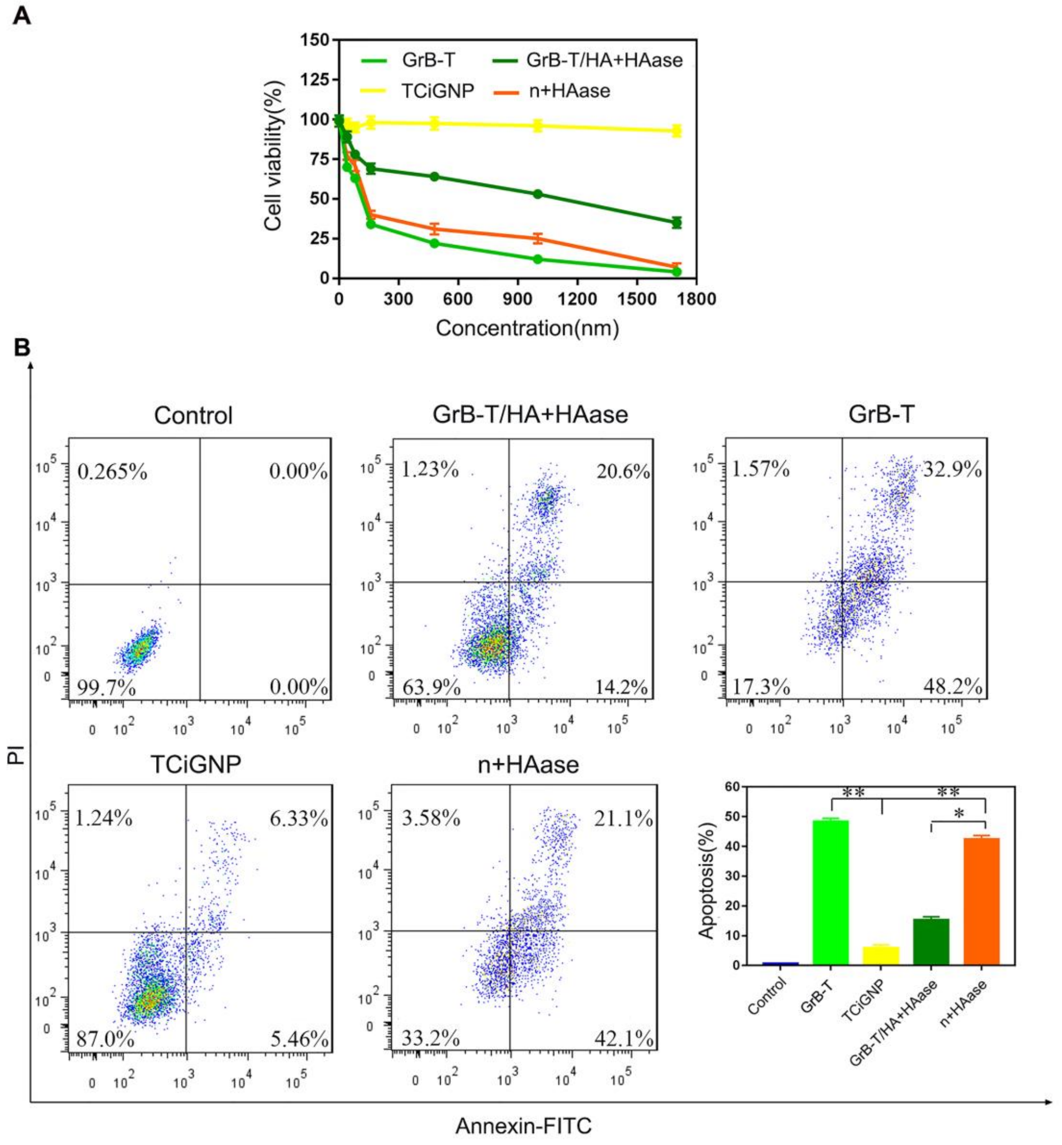

Figure S7. (**A**) Viability of U87 cells treated with different GrB formulations detected by resazurin . (**B**) U87 Cell apoptosis detected by flow cytometry. *P<0.05,**P<0.01.

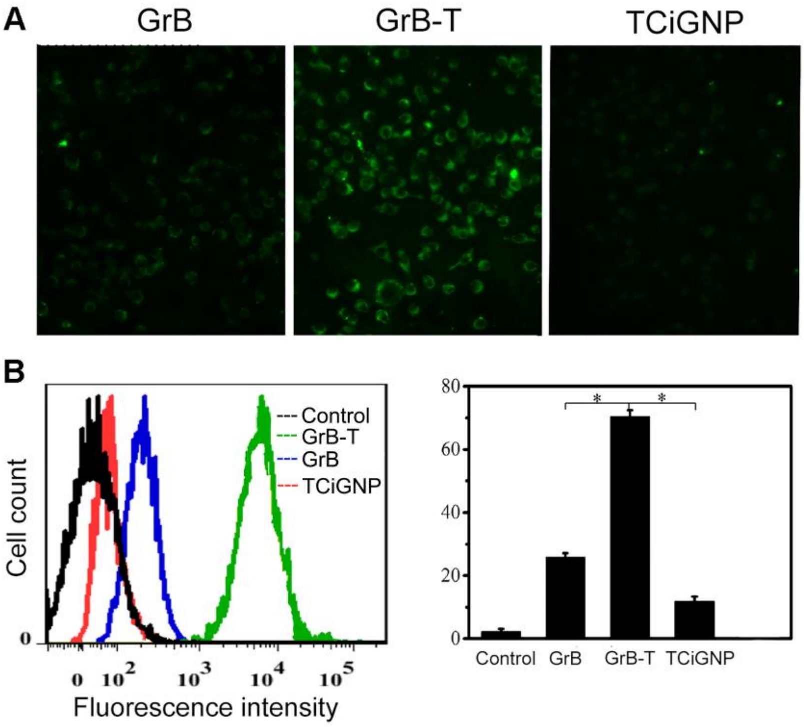

Figure S8. Uptake of the GrB, GrB-T and TCiGNPs in J774A.1 cells by fluorescence microscopy(A) and flow cytometry(B).*P<0.05.

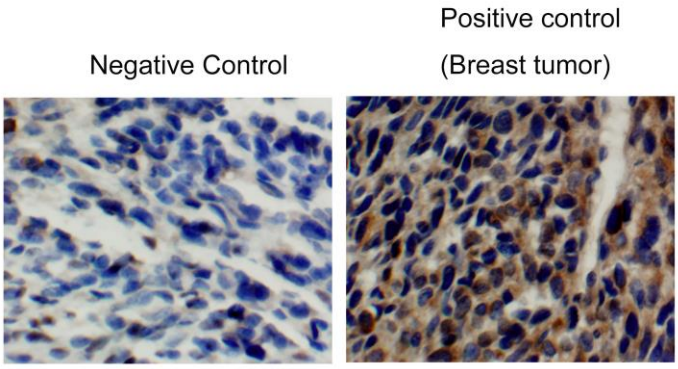

Figure S9. Immunohistochemistry analysis of PDL-1 expression in the tumor tissue . Normal nude mouse tissue was used as a negative control.

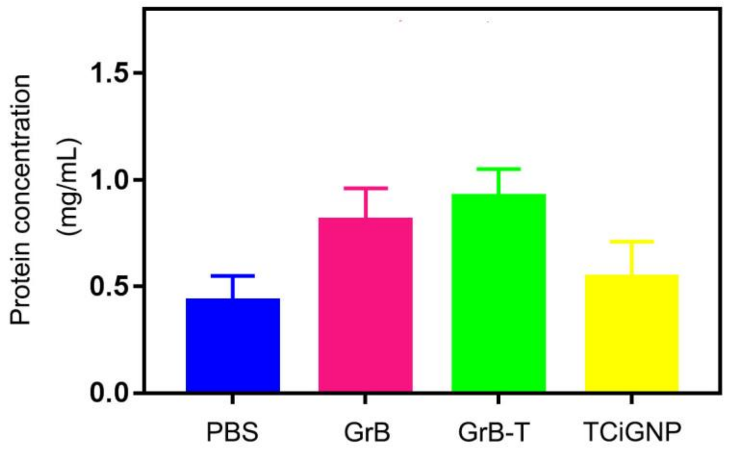

Figure S10. Quantitative measurements of serum proteins adsorbed by TCiGNP and native GrB after incubation with mouse whole serum.

Treatment	Cell line	IC50(nm)	IC50(nm) GrB	Apoptosis (%)	Survival time (Day)
GrB/$VEGF_{121}$[48]	MDA-MB-231	500	2300	---	---
GrB-SPIONs[49]	H1339	---	---	10 (5μg/mL)	39.17
GrB-SPIONs[49]	U87	---	---	8(5μg/mL)	31.7
GrB-CPRPs[50]	A549	20.7	>100	8.01(7.5nm)	48
TCiGNP	MDA-MB-231	131	1750	41.6(100nm/L)	>62
TCiGNP	U87	146	2200	42.1(100nm/L)	---

Table S1. Comparison of GrB IC50, apoptosis, and survival time between TCiGNPs and other reported particles.
